# Elements of Pathological Anatomy

**Published:** 1846-03

**Authors:** 


*Elements of Pathological Anatomy,* illustrated by numerous engravings. By Samuel D. Gross, M. D., Professor of Surgery in the Medical Institute of Louisville, late Professor of General Anatomy, Physiology, and Pathological Anatomy in the Medical Department of the Cincinnati College, Surgeon to the Louisville Marine Hospital, &c. &c. Second Edition, thoroughly revised and greatly enlarged. Philadelphia: Ed. Barrington and Geo. D. Haswell, 1845, pp. 822.

Dr. Gross, in this work, has endeavored to supply the want, as he considers it, of an American production on pathological anatomy, and the consequent general and lamentable ignorance of the profession upon this subject. The expense and difficulty of procuring foreign publications, accounts, no doubt, in a great measure, for deficiency in knowledge in this department of medical science, which, we trust, will be obviated by the present publication.

The Dr. first presents the general principles of the science; then takes up the lesions of each organ and tissue, individually, "indicating their anatomical characters, and, as far as they are known, their diagnostic signs."

The work indicates diligence, observation and research. We take great pleasure in commending it to our professional brethren.

